# Transcranial Magnetic Stimulation–Electroencephalography (TMS-EEG) in Neurosurgery: Unexplored Path Towards Personalized Brain Surgery

**DOI:** 10.3390/jpm14121144

**Published:** 2024-12-09

**Authors:** Martim Oliveira, Sofia Ribeiro, Asfand Baig Mirza, Amisha Vastani, Alba Díaz-Baamonde, Masumi Tanaka, Ali Elhag, Francesco Marchi, Prajwal Ghimire, Feras Fayez, Sabina Patel, Richard Gullan, Ranjeev Bhangoo, Keyoumars Ashkan, Francesco Vergani, Ana Mirallave-Pescador, José Pedro Lavrador

**Affiliations:** 1 Department of Medical Education, Catolica Medical School, Universidade Católica Portuguesa, 1649-023 Oeiras, Portugal; s-maesoliveira@ucp.pt (M.O.); sofiacasal9@gmail.com (S.R.); 2 Department of Neurosurgery, Queen’s Hospital, Barking Havering and Redbridge NHS Trust, London RM7 0AG, UK; asfand.mirza@nhs.net; 3 Department of Neurosurgery, Imperial College Healthcare NHS Trust, London W2 1NY, UK; a.vastani@nhs.net (A.V.); feras.fayez@nhs.net (F.F.); 4 Department of Neurophysiology, King’s College Hospital Foundation Trust, London SE5 9RS, UK; a.diazbaamonde@nhs.net (A.D.-B.); a.mirallave-pescador@nhs.net (A.M.-P.); 5 Department of Critical Care, King’s College Hospital Foundation Trust, London SE5 9RS, UK; masumi.tanaka@nhs.net; 6 Department of Neurosurgery, King’s College Hospital Foundation Trust, London SE5 9RS, UK; ali.elhag@nhs.net (A.E.); francesco.marchi@nhs.net (F.M.); prajwal.ghimire@nhs.net (P.G.); spatel21@nhs.net (S.P.); richardgullan@nhs.net (R.G.); ranj.bhangoo@nhs.net (R.B.); k.ashkan@nhs.net (K.A.); francesco.vergani@nhs.net (F.V.); 7 Department of Neurosurgery, Neurocenter of Southern Switzerland, Ente Ospedaliero Cantonale, CH-6500 Lugano, Switzerland; 8 School of Biomedical Engineering and Imaging Sciences, King’s College London, London WC2R 2LS, UK

**Keywords:** transcranial magnetic stimulation, electroencephalography, neurosurgery

## Abstract

**Background:** Transcranial Magnetic Stimulation–Electroencephalography (TMS-EEG) is a non-operative technique that allows for magnetic cortical stimulation (TMS) and analysis of the electrical currents generated in the brain (EEG). Despite the regular utilization of both techniques independently, little is known about the potential impact of their combination in neurosurgical practice. **Methods:** This scoping review, conducted following PRISMA guidelines, focused on TMS-EEG in epilepsy, neuro-oncology, and general neurosurgery. A literature search in Embase and Ovid MEDLINE returned 3596 records, which were screened based on predefined inclusion and exclusion criteria. After full-text review, three studies met the inclusion criteria. Two independent investigators conducted study selection and data extraction, with mediators resolving disagreements. The NHLBI tool was used to assess risk of bias in the included studies. **Results:** A total of 3596 articles were screened following the above-mentioned criteria: two articles and one abstract met the inclusion criteria. TMS-EEG is mentioned as a promising tool to evaluate tumor–brain interaction, improve preoperative speech mapping, and for lateralization epileptic focus in patients undergoing epilepsy surgery. Lack of detailed patient and outcome information preclude further considerations about TMS-EEG use beyond the potential applications of this technique. **Conclusions:** TMS-EEG research in neurosurgery is required to establish the role of this non-invasive brain stimulation-recording technique. Tumor–brain interaction, preoperative mapping, and seizure lateralization are in the front row for its future applications.

## 1. Introduction

**Figure 1:**
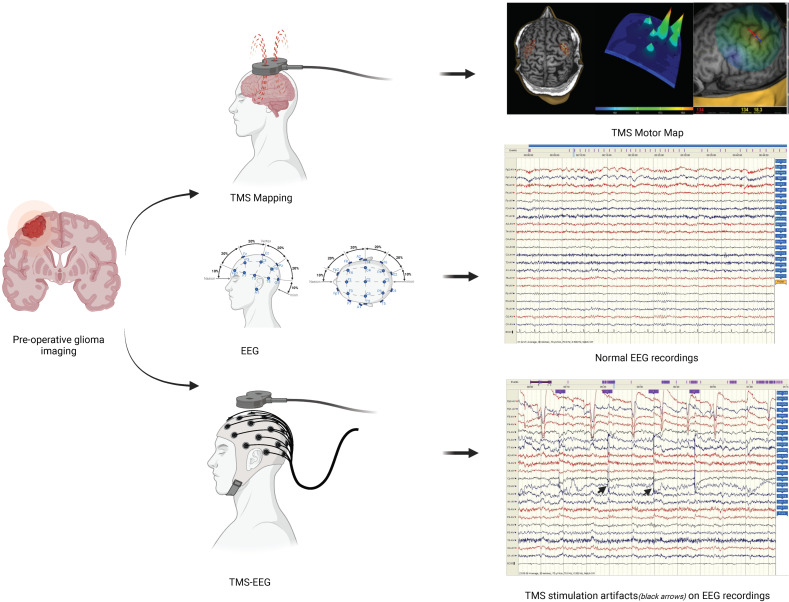
Schematic workflow for TMS-EEG concept—created with BioRender.com.

Navigated transcranial magnetic stimulation with concurrent use of electroencephalography (nTMS-EEG) is a non-invasive neuroimaging technique that combines neuronavigated transcranial magnetic stimulation (nTMS) with electroencephalography (EEG). The integration of both modalities allows for the recording of electrical currents induced by magnetic pulses over a targeted area of the brain making it a valuable tool for the study of brain function and connectivity with high precision and in real time [1] (Figure 1).

The origins of TMS-evoked EEG recordings can be traced back to 1989, when Cracco et al. first documented the technique [2]. In these early experiments, however, EEG data were significantly affected by large artifacts, which hindered the accuracy and interpretation of the recordings. Over the years, as the technology evolved, artifact reduction techniques improved, allowing TMS-EEG to become more reliable and applicable in a wider range of settings. Initially, TMS-EEG was primarily employed in neurophysiology research, but recent advancements have led to its broader application as a diagnostic and therapeutic tool [3]. Modern TMS-EEG protocols now include sophisticated methodologies for minimizing artifacts, making it a preferred approach in contemporary neuroscience research [4], clinical neurology—especially for epilepsy assessment and treatment [3]—, clinical psychiatry [5], and in guiding brain stimulation therapies such as transcranial direct current stimulation (tDCS) and repetitive TMS (rTMS) [1].

In the field of neurosurgery, both nTMS [1,4,6,7,8,9,10,11] and EEG [12,13,14,15,16,17] are already well established, but their combined use remains underexplored, and there are limited data on the efficacy of nTMS-EEG in neurosurgical applications. Recent technological developments in navigated transcranial magnetic stimulation now allow for patient-specific anatomical data to be integrated, resulting in more accurate and effective stimulation targeting [18]. This personalized imaging allowed for the use of nTMS as a preoperative mapping tool when the anatomy is distorted by the presence of a space-occupying lesion [19] and, therefore, expanded its potential to predict intraoperative localization of motor [10,20,21] and speech [22] functions. Despite this, motor and speech nTMS mapping differ in their intraoperative functional predictability—it is higher for motor function [23]. Translational studies comparing direct electrical stimulation, fMRI, tractography, and nTMS for motor mapping have supported nTMS’s accuracy and precision [24,25,26]. EEG is widely used in neurosurgery for epilepsy surgery planning and to assess patients with preoperative seizures secondary to brain tumors [27]. Even though resting-state MRI techniques seem to lead the way in the study of brain connectivity [28,29], the worldwide availability of EEG coupled with a favorable economic profile and less demanding technical requirements [27,30] make this an appealing technique to study TMS-induced connectivity. These characteristics, coupled with evidence demonstrating the benefits of nTMS for postoperative motor rehabilitation and the ability of EEG to enhance the effectiveness of nTMS interventions [31], has sparked renewed interest in exploring the potential of this combined approach in neurosurgery [32,33].

This review aims to explore the novel applications of nTMS-EEG within the neurosurgical field and discusses the future perspectives for this re-emerging technique.

## 2. Methods

### 2.1. Registration and Reporting Standards

**Figure 2:**
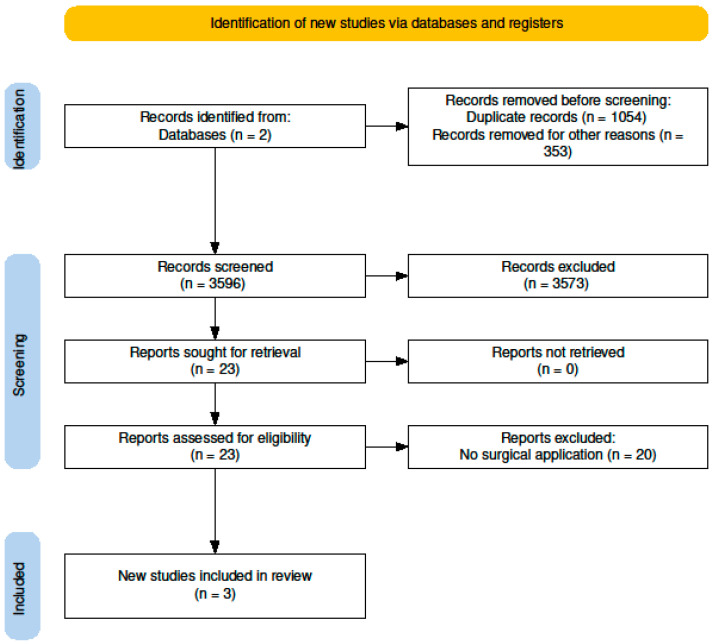
PRISMA flowchart.

We performed this scoping review following the Preferred Reporting Items for Systematic Reviews and Meta-Analyses (PRISMA) guidelines (Figure 2).

#### Protocol Registration

The protocol for this scoping review was registered on the Open Science Framework (OSF) to promote methodological transparency. The registration is currently under embargo while the peer review process is ongoing and will be publicly accessible on 31 December 2024. The protocol can be accessed at https://doi.org/10.17605/OSF.IO/T27KD.

### 2.2. Inclusion Criteria

The inclusion criteria for this review encompassed studies that demonstrated an influence on surgical procedures, whether preoperative, intraoperative, or postoperative, specifically within the contexts of epilepsy surgery, neuro-oncology surgery, or general neurosurgery. Studies utilizing transcranial magnetic stimulation (TMS) or TMS combined with electroencephalography (TMS-EEG) as a modality were also included, provided they involved adult human subjects and were published in English.

### 2.3. Exclusion Criteria

Studies were excluded if they did not meet the specified inclusion criteria. This included studies that lacked relevance to surgical procedures in the preoperative, intraoperative, or postoperative phases, as well as those that did not focus on epilepsy surgery, neuro-oncology surgery, or general neurosurgery. Additionally, studies that did not utilize TMS or TMS-EEG as a modality, involved non-adult or non-human subjects, were published in languages other than English, or addressed non-relevant clinical topics were also excluded.

### 2.4. Search Strategy and Databases

The literature search was performed using Embase and Ovid MEDLINE(R) ALL from inception to the date of search. At the date of search (7 November 2023), our inputs returned 5003 records. After removing duplicates and restricting to the English language and to humans only, we ended up with a total of 3596 records to review according to the defined inclusion and exclusion criteria. Using title and abstract screening, 45 papers were identified for full paper screening. These were found to encompass 22 papers focusing on stroke, 20 on epilepsy, and 3 on general neurosurgery.

**Figure A1:**
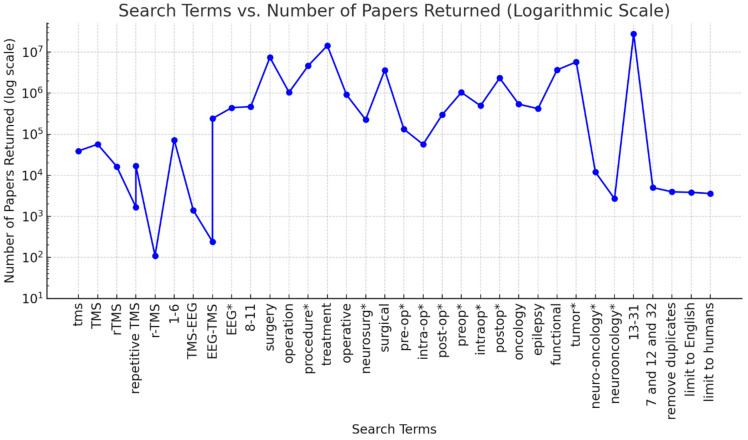
Number of papers returned at each step of search. Please note the * (asterisk) in a search is used as a truncation symbol to find variations of a word that share the same root.

After eliminating all stroke-related papers, 23 full papers were assessed further for surgical application. Of these, 3 met our inclusion criteria and were included in this review. All search terms and the number of papers for each year are included in the Appendix A, Figure A1 and Table A1.

### 2.5. Study Selection

Two investigators (MO and SR) independently screened all titles and abstracts for eligibility. The full text of eligible studies was reviewed for inclusion. ABM and AV acted as mediators in cases of disagreement.

### 2.6. Data Extraction

Data extraction was performed independently by two authors (MO and SR). Data points extracted are displayed in Table 1. Extracted variables were input into an excel spreadsheet for analysis and comparison.

The decision behind proceeding with a scoping and not systematic review rests on the lack of ample information about this topic. Our focus is to map the depth of the existing literature pertaining to the use of TMS-EEG in neurosurgery, identifying key concepts and gaps in knowledge.

### 2.7. Risk of Bias

The NHLBI risk of bias quality assessment tool [34] was used to measure risk of bias in the 3 included items.

## 3. Results

**Figure 3:**
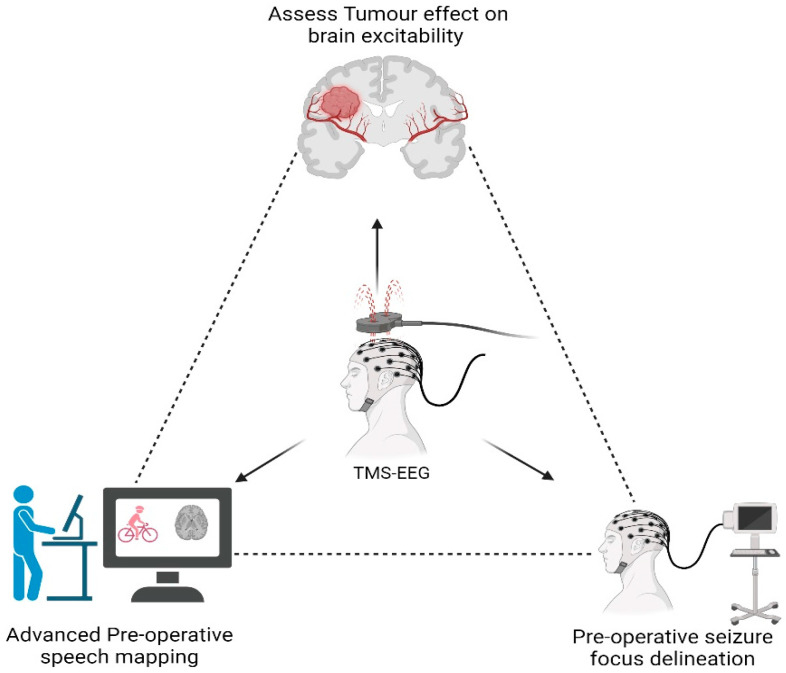
Brain–tumor interface, focus epilepticus detection, and speech mapping—created with BioRender.com.

Out of the 3596 articles initially screened according to the PRISMA (Preferred Reporting Items for Systematic Reviews and Meta-Analyses) criteria, two articles and one abstract met the inclusion criteria for this review. Each study offers unique insights into the use of TMS-EEG in different neurosurgical domains: the impact of tumors on brain excitability, preoperative speech mapping, and the localization of epileptic foci (Figure 3).

### 3.1. Tumor Effects on Brain Excitability

Vucic, S. et al. [35] review the clinical advancements of various TMS techniques, including TMS-EEG, though the article does not exclusively focus on neurosurgical applications. The authors highlight that TMS-EEG can provide a better understanding of how brain tumors influence cortical excitability. This is achieved by analyzing TMS-evoked potentials (TEPs), which are deflections induced in the EEG recordings after a TMS stimulus. The characteristics of these potentials—latency, spread, and directionality—can offer valuable insights into how the tumor affects both the targeted function or tumor-involved brain areas and the surrounding regions. The brain state-dependent effects of TMS suggest that the optimal therapeutic or mapping target may vary based on the patient’s brain state, which can be characterized using EEG. While the paper emphasizes the potential of TMS-EEG in assessing tumor–brain interaction, it does not offer specific metrics or standardized protocols for evaluating tumor-induced changes in brain excitability. This gap in the literature indicates a need for further studies to establish clear guidelines for clinical practice.

### 3.2. Speech Mapping

Lioumis, P. [36] present an abstract proposing a novel approach that combines TMS-EEG for preoperative speech mapping in patients with language-eloquent lesions. This technique aims to enhance mapping precision while potentially reducing the need for awake craniotomy, which is currently the standard for speech mapping in neurosurgery. In this method, TMS is informed by connectivity data from magnetoencephalography (MEG), functional MRI (fMRI), and real-time diffusion-based MRI tractography, and concurrent EEG is used to capture cortical oscillatory activity in response to TMS stimulations. Preliminary findings indicate that TMS-induced errors in speech-related tasks are accompanied by oscillatory EEG signals in language-eloquent regions, while negative stimulations (i.e., those not causing errors) do not produce such activity. Although this approach appears promising for improving the accuracy of preoperative speech mapping, the abstract does not provide specific parameters, such as the stimulation intensity or EEG recording setup. This lack of detailed parameters limits the ability to replicate and generalize the findings across different clinical settings, suggesting that further research is needed to validate the technique and establish standardized protocols for its use in neurosurgery.

### 3.3. Seizure Focus Localization

Rotenberg, A. [37] discuss the wide-ranging clinical potential of TMS-EEG in epilepsy, covering applications from seizure monitoring to therapeutic interventions. Of particular relevance to neurosurgery is the potential use of preoperative TMS-EEG mapping to localize seizure foci in patients undergoing lesionectomy or other epilepsy-related surgeries. This is achieved by identifying epileptiform activity provoked by TMS and recorded via EEG. The study emphasizes the importance of utilizing high-density EEG and co-registration with anatomical imaging (such as MRI) to improve the spatial resolution of TMS-EEG. However, no current data exist comparing the sensitivity, specificity, positive predictive value (PPV), or negative predictive value (NPV) of TMS-EEG to other established methods, such as intracranial EEG or functional imaging, for seizure focus localization. This absence of comparative data highlights the need for further research to evaluate whether TMS-EEG can match or surpass the accuracy of traditional techniques in presurgical planning for epilepsy.

## 4. Discussion

In this review, from the 3596 articles screened, two articles and one abstract were found to meet our inclusion and exclusion criteria, and none of them provided clear patient data and outcome assessment. This argues strongly in favor of further development of this technique in the neurosurgical field. Three topics are mentioned in these results: tumor–brain interaction and preoperative functional mapping with a particular focus on cortical speech mapping and seizure focus lateralization/location in patients eligible for epilepsy surgery. These lay the basis for future research in the TMS-EEG to ascertain the feasibility and widespread implementation within the neurosurgical field.

Assessment of tumor effects on brain excitability and cortical function is crucial for individualized preoperative mapping. There is a different impact of WHO grading and tumor neurobiology on the excitability of the motor cortex [38,39,40,41]. Therefore, preoperative assessment of the tumor effects on brain excitability and cortical function is of great relevance in improving patient counselling, the timing of treatment, preoperative planning, and intraoperative strategies. TMS has been widely used and studied in regard to its preoperative utility. It was shown that an increase in the WHO grading system is associated with an abnormal excitability of the motor-eloquent areas in patients with diffuse gliomas [38], and that nonenhancing motor eloquent gliomas, according to their WHO grading, have a different impact on both the anatomical and functional reorganization of motor areas [39]. In addition, a machine learning model based on TMS-derived interhemispheric excitability is able to provide accurate predictions of high-grade gliomas affecting the motor pathway [40]. Some studies have looked into the effect of tumors on cortical excitability intraoperatively. It was shown that the higher the difference between asleep and awake thresholds in the primary motor cortex, the higher the WHO tumor grading in gliomas [41]. EEG feedback can improve our understanding of preoperative cortical excitability as it has been suggested that EEG-signals from somatosensory cortex (and not from motor cortex) can predict excitability of the corticospinal tract [42]. Also, TMS-EEG can provide valuable preoperative information about preferential patterns of functional connectivity which may improve our understanding of the individual distribution of a specific neurological function and, therefore, potentiate the intraoperative capacity to preserve critical functional hubs [43]. This has potential implications not only during asleep surgery, where the intraoperative functional information at a meta-network level is limited [23], but also during awake surgery where the mapping time needs to be optimized and adapted to each patient, for specific cooperation and engagement in the task assessment [22]. At last, the preservation of function-specific pathways of connectivity as preoperatively identified by TMS-EEG can preserve the scaffold required for postoperative rehabilitation [44] and, therefore, produce better outcomes promoted by rehabilitation strategies [45]. New studies regarding this topic need to provide specific metrics and data that allow for reproducibility of this method and wide application in brain tumor surgery.

Preoperative speech mapping is an ongoing challenge and is more complex when compared to mapping of the functional motor area. A large systematic review [39] was conducted comparing TMS to the current gold standard for speech mapping: intraoperative direct cortical stimulation (DCS). Variability in statistical outcomes was shown, since no guidelines are available for the standardization of cut-offs when determining if a cortical area is considered positive or negative for language function. However, it states that the total number of false-negative-TMS-mapped areas for language function is low in all reviewed studies, and, therefore, the negative predictive value of this technique is high. On the other hand, low PPV and sensitivity are a characteristic of most of the included studies, which means that resection of language-infiltrative lesions based on TMS data alone, without intraoperative DCS, may carry increased functional risks. Multiple studies on speech mapping with TMS have tried different techniques and solutions to improve the results. The proposed solutions fall into two main categories: different patterns of TMS stimulation (such as paired-pulse stimulation [46,47]) and different language tasks during nTMS mapping (such as VAN-POP [48] and CompreTAP [49] tests). The combination of TMS with EEG has the potential to constitute a third approach focused on different recording techniques. TEP analysis enables the speech mapping not only with information regarding the location of the stimulation but also the areas that are affected by the TMS stimulus [50]. This may be relevant when assessing the well-recognized problem of false positive TMS responses [51] according to the presence (versus absence) of certain patterns of TEP response. However, these are early days for this technique as these specific patterns related with true positive responses require definition and intraoperative validation. Nevertheless, preliminary data of the abstract reviewed [50] give a glimpse on how TMS-EEG may be useful, but more studies comparing TMS-EEG with the gold standard DCS should be performed to accurately assess statistical relevance.

Preoperative mapping of seizure lateralization and location of the epileptic focus is challenging. The proportion of patients with epilepsy and drug-refractory epilepsy undergoing surgery has increased. Multiple non-invasive (EEG and its variations, fMRI, single photon emission computer tomography, and magnetoenchephalogram) and invasive (stereo-EEG and subdural grids) methodologies to lateralize and localize epilepsy focus in both lesional and non-lesional epilepsy have significantly increased [52]. Most of them are cumbersome, expensive, and not widely available. In this context, TMS-EEG is a good alternative in multiple ways as it can help with lateralization of the epileptic focus with asymmetry in TMS-induced seizure activity recorded with EEG, and it can provide functional cortical mapping for surgical planning [53]. Cortical functional mapping is significantly different between epilepsy patients and healthy subjects which can be potentially related with the location of the epilepsy focus and epilepsy duration [54]. There is no current data on whether the sensitivity and spatial resolution of TMS-EEG as a modality for presurgical seizure focus localization is meaningful when compared to existing methods. The included study on this topic hypothesized that for a TMS-EEG spatial resolution to be sufficiently enhanced, the utilization of high-density EEG with co-registration of anatomical imaging with EEG responses is needed. Specific patterns of TEP spreading and their correlation with postoperative seizure-related outcomes can improve our understanding and definition of the cortical–subcortical areas requiring resection for the best outcome [55,56]. Future research is required to understand the place of TMS-EEG in epilepsy surgical planning alongside with the well-established interictal imaging [57] and long EEG recordings [58]. However, a technique that is dependent on operator stimulation to induce a predictable response is an appealing alternative to techniques where the observed pathology-specific changes are less predictable.

This review is limited by the lack of the literature available in the neurosurgical field. However, this paper highlights some of the potential main applications of TMS-EEG that should prompt investment and research development by the neurosurgical teams. Other tangential fields to neurosurgery have set the pace in TMS-EEG applications such as stroke recovery patterns [31] and assessment of patients with impaired consciousness [6]. As far as these authors are aware, this is the first paper that attempts to produce a review of the scarce literature on TMS-EEG in neurosurgery and discusses the most promising topics that warrant further research. More comprehensive studies are needed to develop standardized protocols for assessing tumor-induced changes in brain excitability, including specific metrics for evaluating cortical function and tumor interaction. In addition, preoperative speech mapping using TMS-EEG shows promise [1], but further research comparing its efficacy with the current gold standard of direct cortical stimulation (DCS) is crucial to establish its accuracy and safety. In epilepsy surgery, future studies should focus on improving the spatial resolution and sensitivity of TMS-EEG, particularly in localizing seizure foci, by incorporating high-density EEG and advanced imaging techniques. Additionally, other potential applications such as mapping in pediatric neurosurgery, assessing vascular malformations, and optimizing stimulation parameters in deep brain stimulation (DBS) patients should be explored. Expanding research in these areas will help solidify the role of TMS-EEG in neurosurgical practice and potentially improve patient outcomes. This report serves to accentuate this relatively unexplored field rather than providing a conclusive account of clinical results.

## 5. Conclusions

Despite the limited literature exploring TMS-EEG applications within neurosurgery, the findings reviewed highlight its promising potential in enhancing preoperative speech mapping, seizure focus localization, and understanding tumor-induced changes in cortical excitability. These preliminary insights suggest that TMS-EEG can contribute significantly to precision neurosurgery by enabling individualized patient care and optimizing surgical outcomes.

Future research should focus on establishing standardized protocols for TMS-EEG application, particularly in areas such as tumor-induced cortical reorganization, speech mapping validation against gold standards like direct cortical stimulation, and high-resolution seizure focus mapping. Additionally, studies exploring the integration of advanced imaging and machine learning with TMS-EEG could broaden its applicability and refine its clinical utility.

To bridge the gaps in current knowledge, researchers are encouraged to conduct multicenter, longitudinal studies with robust methodological designs to evaluate the sensitivity, specificity, and reproducibility of TMS-EEG across various neurosurgical contexts. Expanding the evidence base will be critical in transitioning TMS-EEG from a promising tool to a standard component of neurosurgical practice.

## Figures and Tables

**Table 1:** Extracted data points from all studies.

Author	Title	Year of publication	Field	Utility of TMS-EEG
Observations	Transcriptions	Mean age	Pathology studied	Number of patients

## Data Availability

No new data was created, the studies in the review are available at the provided references.

## Appendix A

Search terms used:

eeg, eeg-tms, electroencephalogr*, epilepsy, functional, intra-op*, intraop*, neuro-oncolog*, neurooncolog*, neurosurg*, oncology, operation, operative, post-op*, postop*, pre-op*, preop*, procedur*, r-tms, repetitive, tms, transcranial, magnetic, stimulation, rtms, surgery, surgical, tms-eeg, treatment, tumor*.

**Table A1:** Summary and risk of bias.

Paper	Key Findings	Limitations/Risk of Bias
Vucic et al. (2023)—Clinical diagnostic utility of TMS [35]	– TMS is increasingly used to assess brain excitability and map cortical function, particularly in stroke, multiple sclerosis, and motor neuron diseases. – Highlights the potential of TMS to complement neuroimaging techniques. – Calls for more standardized protocols and clinical trials to establish broader diagnostic use.	The review lacks a comprehensive, systematic approach to the literature search and does not clearly define inclusion/exclusion criteria, which may introduce selection bias. There is no mention of dual independent review or assessment of publication bias, increasing the risk of bias in study selection and findings.
Lioumis et al. (2021)—Assessment of Human Cortical Language Network [36]	– TMS-EEG is being explored for non-invasive preoperative speech mapping, reducing the need for awake craniotomies. – Combines TMS with MEG, fMRI, and real-time diffusion MRI tractography to identify language-eloquent regions. – Preliminary results show increased sensitivity and specificity in detecting speech-related cortical activity.	This abstract does not specify eligibility criteria or a systematic search process, which raises the potential for bias. Additionally, the lack of independent review and publication bias assessment increases the risk of biased outcomes.
Rotenberg et al. (2009)—Prospects for Clinical Applications of TMS-EEG in Epilepsy [37]	– TMS-EEG shows promise in mapping cortical excitability and localizing seizure foci for epilepsy surgery. – Could be useful for seizure monitoring and preoperative evaluation. – Current data on TMS-EEG sensitivity and specificity for surgical focus localization is limited; further research is needed.	While the study provides valuable insights, it lacks a rigorous literature review strategy and does not assess publication bias, leading to potential bias in study inclusion and interpretation of results. There is also no mention of independent quality assessment of included studies.
